# Levels of inflammatory markers are differentially expressed in sickle cell anemia and sickle cell trait

**DOI:** 10.1002/jha2.712

**Published:** 2023-05-26

**Authors:** Ingrid Cristiane Pereira Gomes, Lucas Sousa Magalhães, Lays Gisele Santos Bomfim, Priscila Lima dos Santos, Roberto Jerônimo dos Santos Silva, Maria Carollyne Passos Cruz, Lindemberg Costa de Albuquerque, Victoria Haydée Deusdedith Neves, Paula Gabriella Pereira Rosa de Oliveira, Alessandro Freire Carvalho, Lucas Oliveira Silva, Matheus Todt Aragão, Tatiana Rodrigues Moura, Rosana Cipolotti

**Affiliations:** ^1^ Health Sciences Graduate Program Federal University of Sergipe Aracaju Sergipe Brazil; ^2^ Department of Medicine Federal University of Sergipe Aracaju Sergipe Brazil; ^3^ Department of Medicine Tiradentes University Aracaju Sergipe Brazil; ^4^ Laboratory of Molecular Biology and Immunology Federal University of Sergipe Aracaju Sergipe Brazil; ^5^ Sector of Parasitology and Pathology Biological and Health Sciences Institute Federal University of Alagoas Maceió Alagoas Brazil; ^6^ Department of Health Education Federal University of Sergipe Lagarto Sergipe Brazil; ^7^ Department of Physical Education Federal University of Sergipe, São Cristóvão, Sergipe, Brazil São Cristóvão Sergipe Brazil; ^8^ Department of Nutrition Federal University of Sergipe Lagarto Sergipe Brazil; ^9^ Department of Nutrition Tiradentes University Aracaju Sergipe Brazil; ^10^ Department of Morphology Federal University of Sergipe São Cristóvão Sergipe Brazil

**Keywords:** adults, cytokines, inflammation, sickle cell anemia, sickle cell trait

## Abstract

Although sickle cell anemia (SCA) is related to inflammation, the profile of inflammatory markers in sickle cell trait (SCT) is poorly studied. This is a cross‐sectional study of inflammatory biomarkers carried out involving adults with SCA in steady state, SCT and controls. The SCA group had higher levels of lactato dehydrogenase, IL‐1β, IL‐6, IL‐10, and tumor necrosis factor alpha than the others, while the SCT group had similar levels to control group. In addition, SCA group had lower IL‐8/IL‐10 and soluble triggering receptor expressed on myeloid cells‐1/IL‐10 ratios. These findings indicate that individuals with SCT do not have a chronic inflammatory profile and reinforce that cytokines are involved in the maintenance of the inflammatory state in SCA.

## 1 INTRODUCTION

Sickle cell anemia (SCA) is the most prevalent hereditary hemoglobinopathy in the world and is characterized by the production of a variant form of hemoglobin, the S hemoglobin (HbS) [1]. Individuals with SCA are homozygous for HbS, and their erythrocytes assume a sickle shape. Recurrent vaso‐occlusion, the ischemia‐reperfusion process and consequent activation and injury of vascular endothelial cells induce a chronic inflammatory response in the individual with SCA, which is propagated by high levels of inflammatory cytokines, decreased nitric oxide bioavailability and oxidative stress [2]. On the other hand, the heterozygous genotype (Hb‐AS) is sickle cell trait (SCT), considered a benign condition, which morbidity and mortality rates are comparable to the general population's [3]. Even though most individuals with SCT are asymptomatic, studies point out possible complications for this condition, such as hematuria, renal papillary necrosis, hyposthenuria, splenic infarction, exertional rhabdomyolysis, and sudden exercise‐related death [4].

Although much is already known about the involvement of inflammatory mediators in SCA pathophysiology, there is no clear information regarding inflammatory markers in SCT. Therefore, the present study assessed the levels of the inflammatory markers, which were most previously studied in SCA and are associated with inflammatory i (IL‐1β, IL‐6, IL‐8, and tumor necrosis factor alpha [TNF‐α]) and modulator (IL‐10) patterns in non‐infectious inflammatory diseases, in adults with SCT and SCA in steady state.

## 2 MATERIALS AND METHODS

This study is a cross‐sectional controlled study that involved adults with SCA (Hb‐SS Group) age 19 years or older in a steady state followed up at the outpatient Hematology service of the University Hospital of the UFS from March 2019 to January 2021. The exclusion criteria were pregnancy or breastfeeding, blood transfusion in the month prior to collection, previous disorder that led to low bone mass, inflammatory disease, including obesity (BMI≥30 kg/m^2^), systemic use of steroids and/or non‐steroidal anti‐inflammatory drugs in the past 7 days. The SCT group (Hb‐AS Group) was composed of parents of children with SCA who were followed up at the outpatient clinic and other adults with SCT. Healthy individuals matching age and gender with Hb‐SS Group made up the control group (Hb‐AA Group). All participants underwent hemoglobin electrophoresis to confirm the absence of any hemoglobinopathy or HbS in heterozygosis. The exclusion criteria applied to Hb‐SS Group were also applied to the others.

Inflammatory markers were measured in the serum, which was obtained from the peripheral blood of participants and stored at −70°C by an average of 10 months. All the samples were thawed and the cytokines interleukin (IL) ‐ 1β, IL‐6, IL‐8, IL‐10 and the TNF‐α were immediately and simultaneously quantified by multiplex assay using Human Custom ProcartaPlex 5‐Plex Kits (Thermo‐Fisher Scientific, EUA) and the cytokine concentrations were analyzed by MILLIPLEX Analyst 5.1 software (Merck Millipore, Billerica, USA). The soluble triggering receptor expressed on myeloid cells (sTREM‐1) concentrations were performed by a specific enzyme‐linked immunosorbent assay kit (DuoSet, R&D Systems; Abingdon, UK). Lactato dehydrogenase (LDH) was measured using the optimized ultraviolet kinetic method.

### 2.1 Statistical analysis

The data normality analysis was performed using the D`Agostino‐Pearson test. For variation comparisons between groups, the Kruskal–Wallis test was used, followed by the Dunn test for specific differences between groups. GraphPad Prism 5.0 was used to assess the cytokine values. Differences were considered significant if *p* < 0.05.

## 3 RESULTS

Fifty‐five individuals in the Hb‐SS group, 46 individuals in the Hb‐AS group, and 60 individuals in the Hb‐AA group were assessed. Demographic and clinical characteristics of the participants are shown as Supplementary Data (Table 1).

**FIGURE 1:**
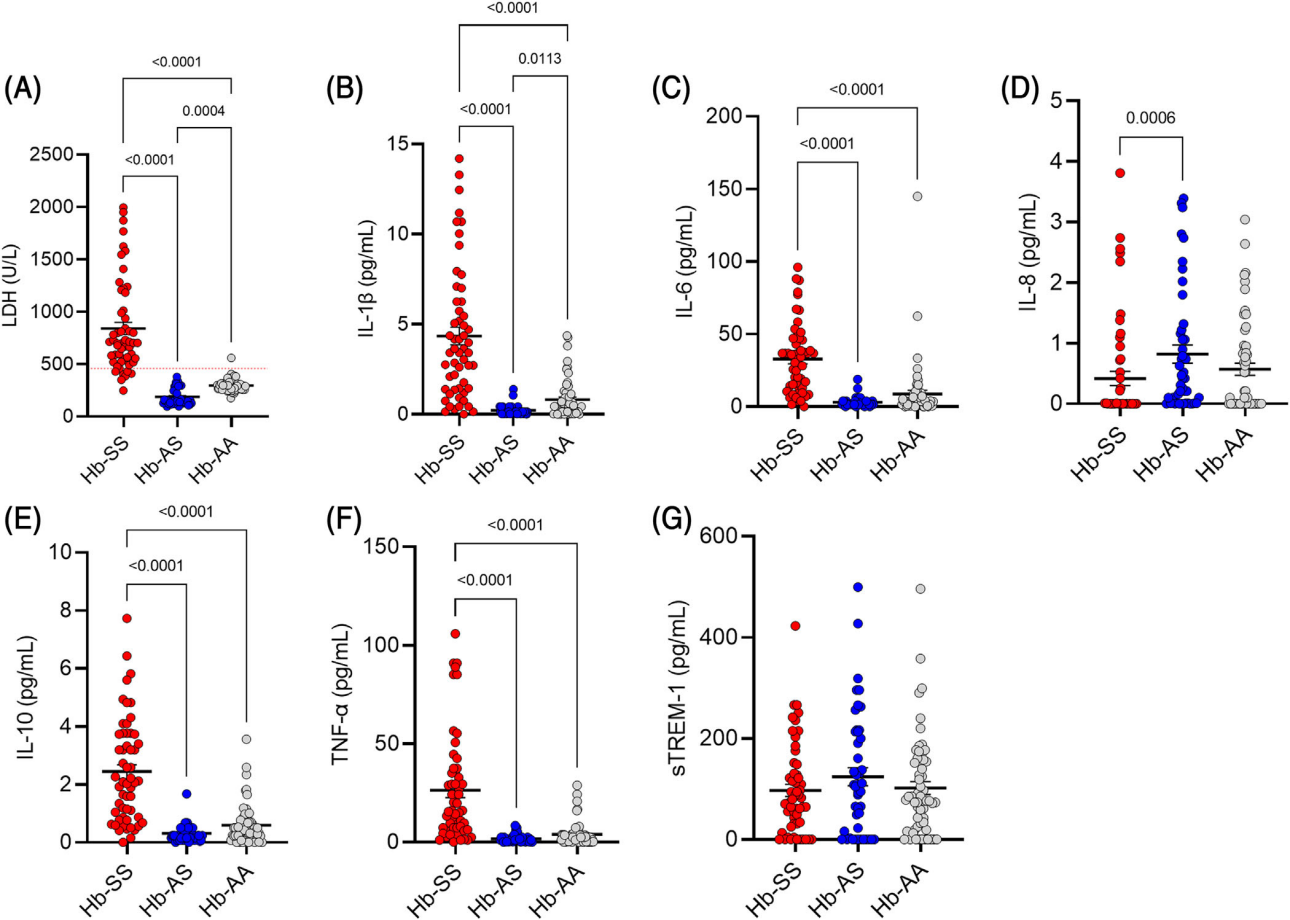
Serum levels of inflammatory markers lactato dehydrogenase (LDH) (1A), IL‐1β (1B), IL‐6 (1C), IL‐8 (1D), IL‐10 (1E), TNF‐α (1F), and sTREM‐ 1 (1G) in the Hb‐SS (red), Hb‐AS (blue), and Hb‐AA (grey) groups. Dots represent each patient included in analysis, and lines represent mean plus standard error of the mean. Differences were assessed using Kruskal–Wallis and Dunn's posttest.

Differences between Hb‐SS, Hb‐AS and Hb‐AA groups were noted for most of the inflammatory markers, except for sTREM‐1. The levels of LDH (Figure 1A), IL‐1β (Figure 1B), IL‐6 (Figure 1C), IL‐10 (Figure 1E), and TNF‐α (Figure 1F) were higher in the Hb‐SS group than the others. The Hb‐SS group had lower IL‐8 levels than the Hb‐AS group (Figure 1D). Comparing the Hb‐AA and Hb‐AS groups, the latter had lower levels of LDH and IL‐1β (Figure 1A and 1B).

**FIGURE 2:**
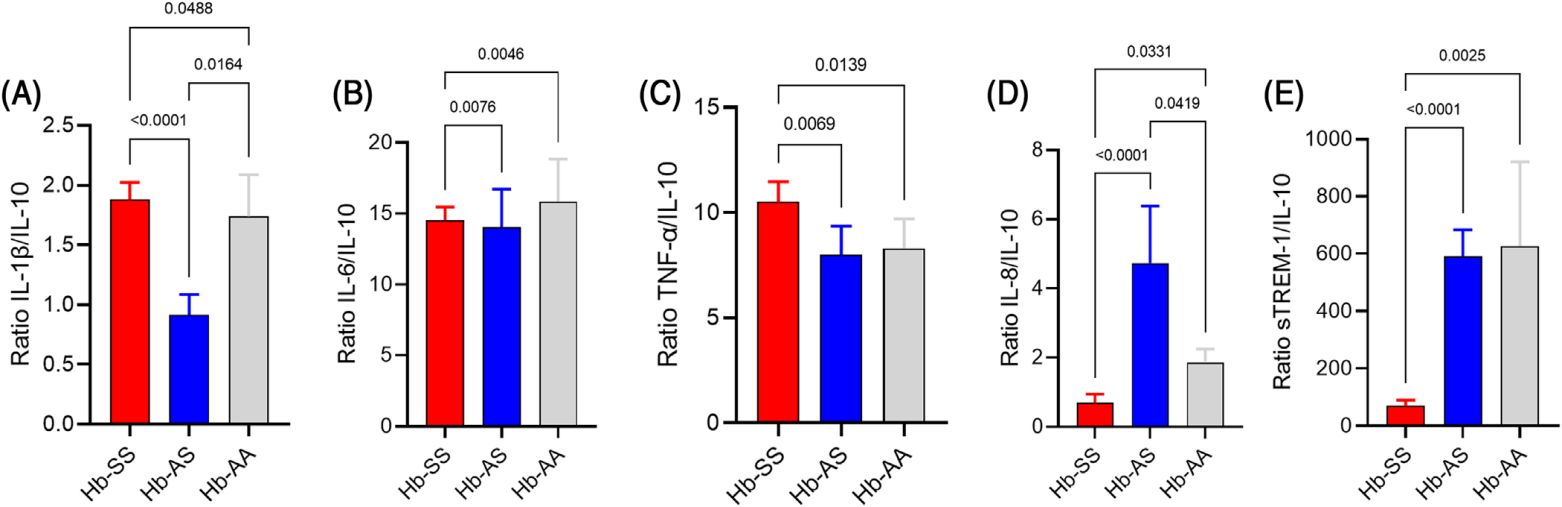
Serum levels of inflammatory cytokines/IL‐10 ratios IL‐1β/IL‐10 (2A), IL‐6/IL‐10 (2B), TNF‐α/IL‐10 (2C), IL‐8/IL‐10 (2D), sTREM‐ 1/IL‐10 (2E) in Hb‐SS (red), Hb‐AS (blue) and Hb‐AA (grey) groups. Bars and upper lines represent mean plus standard error of the mean. Differences were tested using Kruskal–Wallis plus Dunn tests.

From the highest levels of IL‐10 in the Hb‐SS group and in order to estimate quantitatively the relation between the levels of proinflammatory cytokines and the modulator one, the cytokines ratios and sTREM‐1 to IL‐10 of the three groups were assessed, and maintenance of the previous pattern of cytokines IL‐1β (Figure 2A), IL‐6 (Figure 2B), and TNF‐α (Figure 2C) was seen. As for the IL‐8/IL‐10 ratio, there was a lower average in the Hb‐SS group than in the others and a higher level in the Hb‐AS group than in the Hb‐AA (Figure 2D). The sTREM‐1/IL‐10 ratio was lower in the Hb‐SS group (Figure 2E).

## 4 DISCUSSION

Several inflammatory biomarkers are involved in the pathophysiology of SCA, but as far as we know, this is the first study that comparatively assessed these markers in adults with SCA and SCT. This study confirmed the high levels of these markers in patients with SCA in a steady state and its importance is to know the inflammatory immune profile of individuals with SCT, since they are candidates for bone marrow donors from relatives with SCA.

Although SCT is considered a benign condition, study found that it contributes to inflammatory markers and worsen vascular dysfunction in diabetics [5], what could suggest a pro‐inflammatory profile in SCT individuals. Most of scientific literature deals with inflammatory markers in individuals with SCT during exercise and shows no uniform findings regarding the levels of these markers in this context, however, conclude that regular physical activity reduces endothelial activation and vascular adhesion responses in individuals with SCT [6, 7].

The present study confirmed that Hb‐SS individuals have higher levels of LDH, which reflects the pathophysiology of the disease. The Hb‐AS individuals had the lowest levels of LDH among all that corroborates their asymptomatic condition, in addition to ruling out the occurrence of tissue damage linked to it.

The Hb‐SS group also showed higher levels of pro‐inflammatory cytokines IL‐1β, IL‐6, and TNF‐α and anti‐inflammatory IL‐10 than the Hb‐AA group, which is pointed out in the scientific literature. Previous study observed that IL‐1β followed the same pattern as TNF‐α, with higher levels in Hb‐SS than Hb‐AA individuals, as well as in stable Hb‐SS individuals compared to those in crisis [8]. The evidence of higher pro‐inflammatory cytokines in stable Hb‐SS individuals than in those in crisis [9] corroborates both the results of the current study, as it involved Hb‐SS individuals in steady state, and the fact that SCA is a pathology marked by an inflammatory molecule profile, regardless of the individual's clinical conditions. The behavior of IL‐1β in the Hb‐AS group was similar to that of LDH, in that it was the lowest among the three groups.

IL‐6 and TNF‐α were found higher in Hb‐SS group, as in the scientific literature [10]. Study has assessed inflammatory biomarkers in this population, as in steady state as in crisis, and in healthy controls, and have observed higher levels of TNF‐α in Hb‐SS individuals compared to Hb‐AA, as well as in Hb‐SS individuals in steady state in relation to those in crisis, which corroborates the current findings and the presence of chronic inflammation in SCA [11]. TNF‐α showed a behavior similar to that of IL‐6 in the Hb‐AS group, in that it was lower than in the Hb‐SS group and comparable to the Hb‐AA group. The lower levels of the pro‐inflammatory cytokines IL‐1β, IL‐6, and TNF‐α in Hb‐AS individuals when compared to Hb‐SS individuals, in addition to the similarity of their levels to those of Hb‐AA individuals, indicate the absence of an intermediate inflammatory profile in individuals with SCT [8, 12].

TREM‐1 is a receptor constitutively expressed on neutrophils and monocyte subsets, linked to inflammatory response in infections or non‐infectious inflammatory pathologies, such as sepsis and rheumatoid arthritis [13, 14], and it can also be produced in its soluble form (sTREM‐1). Activation of TREM‐1 in neutrophils is related to increased production of pro‐inflammatory cytokines and reduction of anti‐inflammatory cytokines [15], which highlights its pro‐inflammatory role. The levels of sTREM‐1 were similar between the groups, perhaps because the patients were in steady state. There are no previous reports on sTREM‐1 in SCA. It would be valuable to study sTREM‐1 levels in sickle cell disease patients in vaso‐occlusive crisis, and whether this correlates with other measures of severity.

IL‐10 levels were higher in the Hb‐SS group than the others, as previously reported, both in Hb‐SS individuals in crisis or in steady state when compared to healthy individuals [8], that could indicate compensating mechanism. IL‐10 levels in Hb‐AS individuals were lower than in Hb‐SS ones, what, along with similar levels in the Hb‐AA group, corroborates the idea that Hb‐AS individuals do not have a chronic inflammatory state. Based on these results, an analysis of the ratio between inflammatory cytokines and IL‐10 was performed in order to estimate a suppression profile. It was seen that, in Hb‐SS individuals in steady state, the ratios of IL‐1β, IL‐6 and TNF‐α followed the same pattern as the levels of these cytokines in the groups, showing a minor influence of IL‐10 on these cytokines, while the ratios of IL‐8 and sTREM‐1, which are related, respectively, to the recruitment and activation of neutrophils, showed a profile more suppressed by IL‐10. Thus, we could hypothesize that in Hb‐SS individuals in a steady state the role of neutrophils in the chronic inflammatory process is less intense and that this inflammatory state is mediated especially by IL‐1 β, IL‐6 and TNF‐α.

## 5 CONCLUSION

The lack of evidence in the studied sample of an inflammatory profile of the Hb‐AS group in relation to the Hb‐SS and Hb‐AA groups points to the inexistence of a chronic inflammatory condition in these individuals. This fact establishes a positive point regarding the therapy of SCA, since Hb‐AS individuals are potential bone marrow donors whose transplantation is considered the only method of curative treatment to the disease.

## AUTHOR CONTRIBUTIONS

Ingrid Cristiane Pereira Gomes, Tatiana Rodrigues Moura, and Rosana Cipolotti designed the study. Ingrid Cristiane Pereira Gomes, Maria Carollyne Passos Cruz, Priscila Lima dos Santos, Matheus Todt Aragão, Paula Gabriella Pereira Rosa de Oliveira, Alessandro Freire Carvalho, and Victoria Haydée Deusdedith collected detailed data on participants and performed the experiments. Lindemberg Costa de Albuquerque and Lucas Oliveira Silva performed data tabulation. Ingrid Cristiane Pereira Gomes, Lays Gisele Santos Bomfim, Lucas Sousa Magalhães, and Roberto Jerônimo dos Santos Silva analyzed the data. Ingrid Cristiane Pereira Gomes, Lindemberg Costa de Albuquerque, Lucas Sousa Magalhães, and Lays Gisele Santos Bomfim wrote the paper. Ingrid Cristiane Pereira Gomes, Lays Gisele Santos Bomfim, Tatiana Rodrigues Moura, Priscila Lima dos Santos, and Rosana Cipolotti reviewed the paper. All authors reviewed and approved the final version of the paper.

## FUNDING INFORMATION

This publication was supported by the Coordenação de Aperfeiçoamento de Pessoal de Nível Supeperior (CAPES).

## CONFLICT OF INTEREST STATEMENT

The authors declare that they have no conflict of interest.

## ETHICS STATEMENT

The project was approved by the Ethics Committee in Research Involving Human Beings of the Federal University of Sergipe (CEP HU/UFS), through serial number 2.897.835. Individuals who expressed their consent by appreciating and signing the Free and Informed Consent Term participated in the study.

## Supporting information

Supporting Information

## Data Availability

The data that support the findings of this study are available from the corresponding author upon reasonable request.
