# Supplementary material for: Levels of inflammatory markers are differentially expressed in sickle cell anemia and sickle cell trait
Source: EJHaem. 2023 May 26;4(3):705–9. doi: 10.1002/jha2.712 (PMC10435695; doi:10.1002/jha2.712)
Supplement: Supplementary file 1 — Supporting Information [file JHA2-4-705-s001.docx]

**SUPPLEMENTARY DATA**

| **Table 1:** Demographic and clinical characteristics of adults with sickle cell anemia, sickle cell trait and controls | | | | | | | | | |
| --- | --- | --- | --- | --- | --- | --- | --- | --- | --- |
| Variables | Hb-SS Group | | | Hb-AS Group | | | Hb-AA Group | | |
|  | N (%) | Mean (SD) | Median | N (%) | Mean (SD) | Median | N (%) | Mean (SD) | Median |
| Sex  Female | 29 (52.7) |  |  | 32 (69.6) |  |  | 30 (50.0) |  |  |
| Age |  | 26.5 (5.2) | 26.0 |  | 33.1 (6.9) | 34.0 |  | 28.0 (4.9) | 28.0 |
| Weight (Kg) |  | 56.6 (8.2) | 56.4 |  | 66.3 (9.9) | 66.4 |  | 68.9 (±11.8) | 67.0 |
| Height (cm) |  | 164.4 (8.0) | 164.0 |  | 164.9 (8.0) | 165.0 |  | 169.0 (8.8) | 169.0 |
| BMI (kg/m2) |  | 20.8 (2.5) | 20.5 |  | 24.4 (3.3) | 24.3 |  | 23.9 (2.7) | 23.8 |
| Hb (g/dL) |  | 9.1 (1.6) | 9.0 |  | 14.1 (1.2) | 14.0 |  | 14.5 (1.6) | 14.1 |
| Leukocytes (/mm3) |  | 10444 (4255) | 10100 |  | 6085 (1949) | 5948 |  | 6439 (1592) | 6123 |
| Neutrophils (/mm3) |  | 4941 (2424) | 4805 |  | 3441 (1656) | 3185 |  | 4081 (4675) | 3406 |
| BMI: body mass index; Hb: hemoglobin; SD: standard deviation | | | | | | | | | |
